# Effect of cold plasma on essential oil content and composition of lemon verbena

**DOI:** 10.1002/fsn3.876

**Published:** 2019-02-22

**Authors:** Mohammad‐Taghi Ebadi, Soleiman Abbasi, Amir Harouni, Fatemeh Sefidkon

**Affiliations:** ^1^ Faculty of Agriculture Department of Horticultural Science Tarbiat Modares University (TMU) Tehran Iran; ^2^ Faculty of Agriculture Department of Food Science and Technology Tarbiat Modares University (TMU) Tehran Iran; ^3^ Medicinal Plants Research Division Research Institute of Forests and Rangelands (RIFR) Tehran Iran

**Keywords:** aromatic plant, citral, cold plasma, essential oil, *Lippia citriodora* Kunth.

## Abstract

Cold plasma is known as a novel nonthermal processing method for decontamination of medicinal and aromatic plants (MAPs); however, there are little research studies about its effects on active ingredients of these plants. The aim of this research was to investigate the influence of low‐pressure cold plasma (LPCP) treatments (1, 3, and 5 min) on the essential oil (EO) content and composition of lemon verbena leaves. The EO content was determined using hydro‐distillation, and the composition of the extracted EOs was quantified using gas chromatography and gas chromatography–mass spectrometry techniques. The results showed that by increasing the LPCP treatment duration, the EO content was reduced from 1.2 to 0.9 (% v/w). The highest content of monoterpene hydrocarbons (e.g., limonene) and oxygenated sesquiterpenes (e.g., spathulenol and globulol) was also observed in LPCP‐treated ones, whereas the oxygenated monoterpenes (e.g., citral) content of control was measurably higher than those treated with LCPC.

## 1 INTRODUCTION

Lemon verbena (*Lippa citriodora* Kunth.) is a shrub with scented leaves, from the family Verbenaceae, which grows in tropical and subtropical regions. The leaves of lemon verbena are utilized as seasoning for food preparations and also for flavoring beverages. Historically, the leaves been used for their strong scent as an insect repellant (Ebadi, Azizi, Sefidkon, & Ahmadi, 2015; Pascual, Slowing, Carretero, Mata, & Villar, 2001).

Medicinal and aromatic plants are traded worldwide and usually consist of dried plant parts, which are naturally contaminated. Contamination with microorganisms is one of the most common problems of spices (Thongphasuk & Thongphasuk, 2012). Many spices are cultivated and harvested in poor sanitary conditions, laying the groundwork for potential microbial contamination (McKee, 1995). Also, the initial microbial load of spices depends on the conditions of production, harvesting, and initial processing, such as drying (Ebadi, Sefidkon, Azizi, & Ahmadi, 2016; Hertwig, Reineke, Ehlbeck, Erdoğdu, et al., 2015). Some microorganisms are known human pathogens, requiring methods of disinfection which minimize potential damage to active substances. It should be noted that the use of contaminated spices can reduce the longevity of their by‐products and may even cause health risks to consumers (Hertwig, Reineke, Ehlbeck, Erdoğdu, et al., 2015; Little, Omotoye, & Mitchell, 2003; McKee, 1995).

Currently, various decontamination technologies such as gamma irradiation, heating (steam sterilization), and fumigation (ethylene oxide) have been used to overcome mentioned damages although they have some disadvantages too. For instance, irradiation with gamma rays is an efficient method, but is not easily accepted by consumers. Fumigation by ethylene oxide is also banned in many countries due to having carcinogenic effects in humans. As the same, steam sterilization also has some undesirable effects on active substances of spices, especially in terms of essential oil (EO) content and composition. Therefore, scientists have been looking forward to find new decontamination methods of spices which do not have the drawbacks and limitations listed (Hertwig, Reineke, Ehlbeck, Knorr, & Schlüter, 2015; Kim, Lee, & Min, 2014; Schweiggert, Carle, & Schieber, 2007).

Plasma is the fourth state of matter and resembles an ionized gas. Plasmas can be divided into two groups, based on the method of generation: cold (nonthermal) plasma and thermal plasma (Méndez‐Vilas, 2013). Cold plasma can be produced under vacuum or atmospheric conditions and is named low‐pressure cold plasma (LPCP) and atmospheric cold plasma (ACP), respectively (Hertwig, Reineke, Ehlbeck, Erdoğdu, et al., 2015; Thirumdas, Sarangapani, & Annapure, 2015). Due to the properties of cold plasma, it is used in several fields and the application of this technology as a decontamination tool has been commercially accepted for sterilization of heat‐sensitive medical devices and thermolabile materials used in biomedical technology (Roth, Nourgostar, & Bonds, 2007; Thirumdas et al., 2015). Disinfection by cold plasma technology is due to certain reactive species, such as free radicals, charged particles, and UV photons (Harouni & Abbasi, 2017). This novel technology has the capability to be used for microbial decontamination of spices. It is a nonthermal method and enables the elimination of vegetative bacteria, bacterial endospores, molds, and yeasts under nonthermal conditions (Hertwig, Reineke, Ehlbeck, Erdoğdu, et al., 2015; Kim et al., 2014). Several studies have been reported on decontaminating effect of cold plasma on agricultural products (Baier et al., 2013; Basaran, Basaran‐Akgul, & Oksu, 2008; Jahid, Han, & Ha, 2014; Kim & Min, 2017; Lacombe et al., 2015; Lee, Kim, Chung, & Min, 2015; Misra et al., 2014; Niemira & Sites, 2008; Ouf, Basher, & Mohamed, 2015; Tappi et al., 2016), and on spices in particular (Grabowski, Strzelczak, & Dąbrowski, 2014; Hertwig, Reineke, Ehlbeck, Erdoğdu, et al., 2015, 2015; Hertwig, Reineke, Ehlbeck, Knorr, et al., 2015, 2015; Kim, Oh, Won, Lee, & Min, 2017; Kim et al., 2014). However, information about its impact on the quantity and quality of the active ingredients is limited. Lemon verbena is of agricultural origin and so is usually highly contaminated by microorganisms before any decontamination processes. Therefore, in this study, the influence of LPCP on the EO content of lemon verbena leaves, as well as its composition, was examined.

## 2 MATERIALS AND METHODS

### 2.1 Plant material

Lemon verbena shrubs were cultivated in greenhouse at Tarbiat Modares University, Tehran, Iran. Then, the leaves were carefully harvested in the flowering stage and dried for 72 hr under shade conditions at room temperature (20–25°C).

### 2.2 Plasma supply and treatment

**Figure 1:**
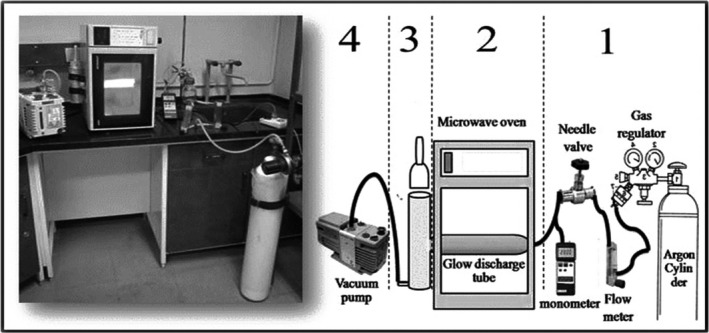
Illustration of a low‐pressure cold plasma (LPCP) generator designed at Tarbiat Modares University actual (left) and schematic (right) settings (Harouni & Abbasi, 2017)

The LPCP system, designed by academic faculty group of TMU (Figure 1), was composed of a microwave generator (domestic microwave oven, 1200W, 2.45 GHz; AEG Micromat, Nürnberg, Germany), a gas pressure and flow rate controller (Model DK800‐04; Arshia Engineering Company, Tehran, Iran), a treatment chamber (Pyrex, 75 mm diameter and 210 mm length), and a vacuum pump (Edward RV3, England). The inner pressure of the plasma reactor was monitored by a vacuum metering device (Model PM‐9107; Lutron, Taiwan), and the feeding gases were nitrogen (N2), argon (Ar), and oxygen (O_2_). Ten grams of the dried lemon verbena leaves was placed in the reaction chamber (glass tube with a volume of 333.82 cm^3^) and treated by LPCP in different time periods, namely 0 (control), 1, 3, and 5 min. Each treatment was repeated at least three times (Harouni & Abbasi, 2017).

### 2.3 Essential oil extraction

Three dried samples each weighing 20 g were hydro‐distilled for 3 h using the Clevenger apparatus. EO content was determined on the basis of dry matter (ml/100 D.M.). The removal of moisture from EOs was performed by adding 0.5 g sodium sulfate and then keeping in dark vials at 4°C until the time of analysis (Dianat, Saharkhiz, & Tavassolian, 2016).

### 2.4 Gas chromatography

Gas chromatography (GC) analysis was carried out with a Thermo‐UFM (Ultra‐fast model, Italy) gas chromatograph equipped with flame ionization detector (FID) and a Ph‐5‐fused silica column (10 m × 0.1 mm; 0.4 μm film thickness). The oven temperature was held constant at 60°C for 5 min and then programmed a rate of 80°C/min to 285°C. The carrier gas was helium with a flow rate of 0.5 ml/min; injector and detector temperatures were 280°C. The percentages of compounds were measured making use of the area normalization method, without considering response factors (Sefidkon, Abbasi, Jamzad, & Ahmadi, 2007).

### 2.5 Gas chromatography–mass spectrometry

Gas chromatography–mass spectrometry (GC/MS) analysis was conducted with a Varian 3400 GC connected to a mass spectrometer Saturn model, equipped with a DB‐5‐fused silica capillary column (30 m length, 0.25 mm internal diameter and 0.25 μm film thickness); the injection chamber temperature and the transfer line were set at 260 and 270°C, respectively. The carrier gas was helium with a linear velocity of 31.5 cm/s, split ratio of 1 to 60, 1 s of scan time, and mass range of 40–300 a.m.u. The oven temperature adjustment program was the same as the one mentioned for GC. The constituents of the EO were determined by calculating their retention indices and comparing related mass spectra with those registered in a computer library or the ones reported in the literature. To meet quantification purposes, relative area percentages obtained by FID were used without the use of correction factors (Adams, 2007; Davies, 1990; Sefidkon et al., 2007).

### 2.6 Data analysis

The experiments were conducted based on completely randomized design (CRD) with three replications. The analysis of variance and mean comparisons of data were performed using Duncan's multiple range test at 5% significance level, and the statistical analysis of the results was done by SAS 9.1 software.

## 3 RESULTS AND DISCUSSION

### 3.1 Effect of cold plasma treatment on EO content

**Figure 2:**
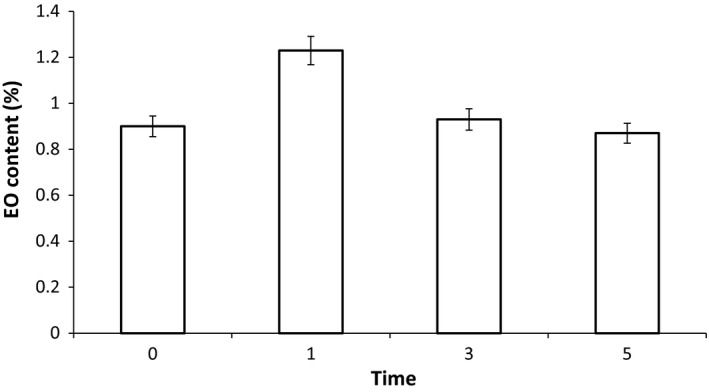
Influence of LPCP treatments on EO content (V/W %) of lemon verbena. EO: essential oil; LPCP: low‐pressure cold plasma

The results of the EO content analysis in different LPCP treatments are presented in Figure 2. As it can be seen, the LPCP had a significant effect on EO content (*p* < 0.05). For example, after 1 min of LPCP treatment, the EO content increased by 36.7% compared to the control (0.9 ml/100 g D.M.); however, the 3‐ and 5‐min treatments (0.93 and 0.87 ml/100 g D.M., respectively) did not show any significant difference from control. In other words, by extending LPCP treatment duration from 1 to 5 min, EO content was significantly decreased. These results were in accordance with other researchers observations. For instance, Kodama, Thawatchaipracha, and Sekiguchi (2014) reported that plasma treatment increased the EO yield of lemon peel, while longer plasma treatment time boosted the evaporation of EO components during the treatment, which resulted in lower amounts of EOs. Cold plasma treatment may cause changes in the physical surface properties of the product that leads to porosity. Scanning electron microscopy showed the formation of fissures and depressions on the surface of product after exposure to cold plasma. These surface changes are believed to provide routes for faster EO evaporation (Kodama et al., 2014; Mir, Shah, & Mir, 2016; Misra, Schlüter, & Cullen, 2016).

### 3.2 Effect of cold plasma treatment on EO composition

**Figure 3:**
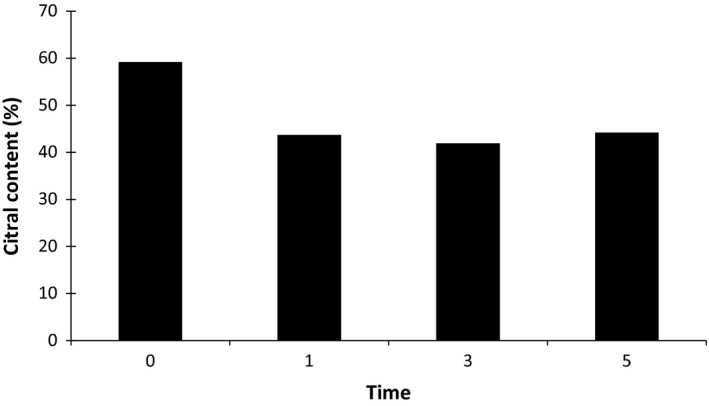
Citral (geranial + neral) content (%) of lemon verbena EO affected by LPCP. EO, essential oil; LPCP: low‐pressure cold plasma

Based on our observations, 24 different compounds were recognized in the EOs of *L. citriodora* which were affected by LPCP treatments. The chemical compounds of the EOs are shown in Table 1. The main constituents of the EO in all treatments were geranial, neral, limonene, 1,8‐cineole, and globulol. It was revealed that LPCP treatments caused some variation in the main components of EO that alters the chemical profiles of lemon verbena EO (Table 1 and Figure 3). The greatest concentration (59.2%) of citral (geranial + neral) was observed in the control samples, and the lowest amount (41.9%) was measured in the samples treated for 3 min of LPCP. Furthermore, no significant difference was observed on other LPCP treatments (43.7% and 44.2% for 1‐ and 5‐min LPCP treatments, respectively). Apart from citral, LPCP treatments also showed some effects on other compounds. For example, the limonene, 1,8‐cineole, and γ‐elemene contents were slightly increased (7.3%, 2.6%, and 2.9% compared to the control) after 3 min of LPCP treatment. The maximum amounts of spathulenol and globulol were observed after 5 min of LPCP treatment (8.1% and 7.3%, respectively).

**Table 1:** Comparison of the chemical composition of lemon verbena EOs before and after LPCP treatment

No.	Compound	RI^a^	Duration of LPCP (min)			
			0	1	3	5
1	α‐Pinene	938	—	0.4	0.3	0.3
2	Camphene	953	0.5	—	—	—
3	Sabinene	981	2.4	1.4	1.2	1.3
4	Limonene	1,028	5.8	12.6	13.1	11.5
5	1,8‐Cineole	1,031	6.2	8.6	8.8	8.2
6	γ‐Terpinene	1,062	0.4	0.5	—	0.5
7	Terpinolene	1,090	0.6	0.2	0.3	—
8	Transpinocarveol	1,140	0.3	—	—	—
9	*cis*‐sabinol	1,143	0.7	—	—	—
10	Citronellal	1,152	0.7	1.1	1.0	1.1
11	α‐Terpineol	1,190	0.7	1.2	1.0	1.1
12	Nerol	1,228	0.2	0.3	—	—
13	Neral	1,238	23.3	18.4	17.3	18.7
14	Geranial	1,267	35.9	25.3	24.6	25.5
15	Neryl acetate	1,360	1.4	1.0	0.9	1.1
16	α‐Copaene	1,379	0.5	0.3	—	—
17	α‐Gurjunene	1,410	0.2	—	—	—
18	E‐caryophyllene	1,421	1.0	0.5	0.5	0.8
19	γ‐Elemene	1,439	5.0	7.1	7.9	7.6
20	α‐Humulene	1,456	0.6	0.4	1.0	0.4
21	Cubenol	1,514	1.6	—	—	0.3
22	Spathulenol	1,580	5.2	7.3	7.7	8.1
23	Globulol	1,587	3.7	6.8	6.8	7.3
24	Epi‐α‐cadinol	1,642	0.5	0.7	0.9	0.7
	Total		97.4	94.1	93.3	94.5

*Notes*

EO, essential oil; LPCP, low‐pressure cold plasma.

a Retention index.

**Figure 4:**
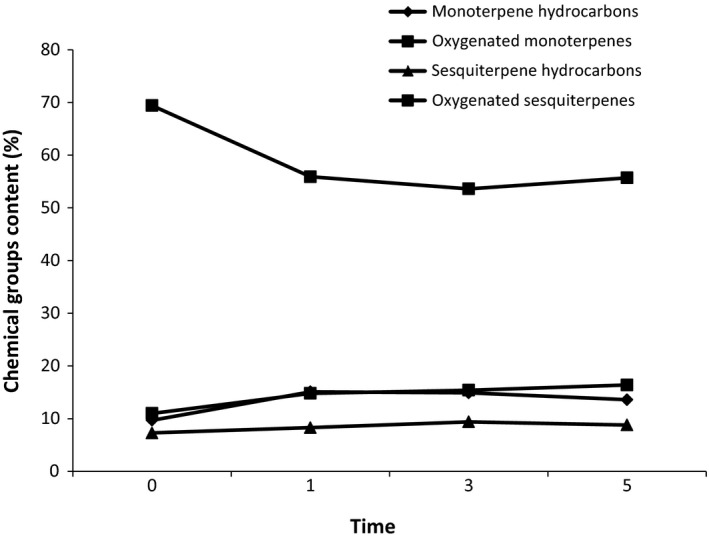
Chemical groups (%) of lemon verbena EO affected by LPCP. EO, essential oil; LPCP: low‐pressure cold plasma

The constituents of lemon verbena EOs in all treatments could be classified into these following chemical groups (Figure 4): monoterpene hydrocarbons (9.7%–15.1%), oxygenated monoterpenes (53.6%–69.4%), sesquiterpene hydrocarbons (7.3%–9.4%), and oxygenated sesquiterpenes (11%–16.4%). The highest amount of oxygenated monoterpenes (69.4%) was observed in the control samples, but the maximum amount of other chemical groups was obtained for LPCP‐treated samples. In other words, the effectiveness of LPCP on EO was demonstrated in terms of its quality and quantity. Kodama et al. (2014) reported that plasma treatments had a significant effect on citrus EO composition, and limonene, γ‐terpinene, and β‐pinene in the EO were reduced. It was observed that the highest amount of main compounds was obtained in 1‐min plasma treatment. The oxidation and damaged oil glands were identified as the main reasons for this phenomenon. Hertwig, Reineke, Ehlbeck, Knorr, et al. (2015) reported that the amount of piperine in cold plasma‐treated black pepper was only slightly lower than the amount determined in the untreated one. The results revealed that pepper quality was relatively unaffected by cold plasma treatments (after 15 and 30 min). It seems our findings are in line with the existing studies about the impact of cold plasma (either positive or negative) on the quality of agricultural products (Bußler et al., 2015; Garofulić et al., 2015; Grabowski et al., 2014; Herceg et al., 2016; Hertwig, Reineke, Ehlbeck, Erdoğdu, et al., 2015; Kovačević et al., 2016; Lacombe et al., 2015). These considerable outcomes could be attributed to the differences in plant species, type, and duration of cold plasma treatment, the feed gas used, the applied voltages, plant secretory structures, and chemical composition of active substances (Mir et al., 2016).

## 4 CONCLUSION

Plasma technology is a novel and effective nonthermal decontamination method that can decrease the negative effects of thermal methods on quality characteristics of spices. Upon literature reviews, very few investigations have been focused on the effect of cold plasma on spices active substances. The results of this research revealed that brief LPCP treatment had a positive effect on EO content of lemon verbena leaves, but it reduced the amount of citral while the changes in other compounds were not significant.

## CONFLICT OF INTEREST

The authors declare that they do not have any conflict of interest.

## ETHICAL REVIEW

This study does not involve any human or animal testing.

## INFORMED CONSENT

Written informed consent was obtained from all study participants.
